# Bibliometric analysis of single-cell sequencing researches on immune cells and their application of DNA damage repair in cancer immunotherapy

**DOI:** 10.3389/fonc.2023.1067305

**Published:** 2023-01-26

**Authors:** Yu Zhao, Yuanxun Xiao, Zhengbo Hu, Ji Wang, Zhiguang Xu, Yizhang Mo, Guojun Qi, Kebing Chen, Wu Wu, Weiying Ma

**Affiliations:** ^1^ Department of Hematology, The Third Affiliated Hospital of Southern Medical University, Guangzhou, Guangdong, China; ^2^ Department of Burn & Plastic Surgery, Yuebei People’s Hospital Affiliated to Shantou University Medical College, Shaoguan, Guangdong, China; ^3^ Department of Orthopaedics, Yuebei People’s Hospital Affiliated to Shantou University Medical College, Shaoguan, Guangdong, China; ^4^ Department of Spine Surgery, The Sixth Affiliated Hospital of Sun Yat-sen University, Guangzhou, Guangdong, China; ^5^ Guangdong Provincial Key Laboratory of High Technology for Plant Protection, Plant Protection Research Institute, Guangdong Academy of Agricultural Science, Guangzhou, Guangdong, China; ^6^ Orthopedics Rehabilitation Department, Guangdong Work Injury Rehabilitation Center, Guangzhou, Guangdong, China; ^7^ Department of Anesthesiology, Sun Yat-sen Memorial Hospital, Sun Yat-sen University, Guangzhou, Guangdong, China

**Keywords:** single-cell sequencing, immune cells, T cell, bibliometric analysis, DNA damage repair, immunotherapy

## Abstract

**Introduction:**

In recent decades, single-cell sequencing technology has developed rapidly and used widely in various fields of life sciences, especially for the detection of immune cells. A bibliometric analysis of single-cell sequencing research work on immune cells published during the 2011-2021 period should provide new insight on the use of single-cell sequencing.

**Methods:**

We screened 1,460 publications on single-cell sequencing on immune cells according to the publication date, article type, language, and country.

**Reults:**

The United States published the first and largest number of articles, while China’s research started relatively late, but ranked second in the number of publications. T cells were the most commonly studied immune cells by single-cell sequencing, followed by mononuclear macrophages. Cancer biology was the most common field of immune cell research by single-cell sequencing. Single-cell sequencing studies using γδ T cells were mainly in the fields of cancer biology and cell development, and focused over time from cell surface receptor to cell function. Through in-depth analysis of the articles on single-cell sequencing of T cells in the oncology field, our analysis found that immunotherapy and tumor microenvironment were the most popular research directions in recent years.

**Discussion:**

The combination of DNA damage repair and immunotherapy seems to provide a new strategy for cancer therapy.

## 1 Introduction

Single-cell sequencing technology refers to sequencing genetic information at a single cell level to obtain gene sequences, transcripts, epigenetics, protein expression profiles at a molecular level, identify cell population differences, and cellular evolution (1). Currently, single-cell sequencing is widely used in various clinical fields, such as cancer microenvironment, immunotherapy, embryonic development, and the pathogenesis of cardiovascular disease (2).

In the past, routine methods for detection of immune cells, including flow cytometry, immunofluorescence staining, MHC/polypeptide tetramer method, had certain significance for the diagnosis, therapeutic effect, and assessment of prognosis in certain clinical conditions (3, 4). However, due to the heterogeneity between immune cells, the genome, transcriptome, epigenome and proteome of a single cell cannot be analyzedusing these methods (5).

The publication of the first article on the immune response of CD8 lymphocytes to respiratory disease infection in 2011 by the pioneer American scientist Surman and coworkers (6) opened up the single-cell sequencing technique in the field of immunity. Single-cell sequencing does not only reveal the specificity and function of individual immune cells and study the corresponding molecular mechanisms, but also analyzes immune cell subsets and intercellular networks, explores the mechanisms and differences in the immune system, and discovers the potential functions of immune cells (3, 7, 8). T cells are the most studied immune cells by single-cell sequencing, including the role of T cells in parasitic and viral infections (9, 10), autoimmune diseases (11), and cancer biology (12). γδ T cells are relatively small populations of T cells, but they play an important role (13) such as anti-cancer role directly and indirectly by lysing cancer cells (14–16). Furthermore, γδ T cells can recognize target cells without MHC restriction and play a key role in cancer immune surveillance (17).

The aim of this communication was to conduct bibliometric analysis and knowledge visualization of the currently published scientific papers on single-cell sequencing technology using immune cells, especially T cells and γδ T cells. The hotspots of application are reviewed in order to provide the basis for follow-up research.

## 2 Materials and methods

### 2.1 Source database

We used the Scopus database (http://www.scopus.com/) for the search. The Scopus database is the world’s largest peer-reviewed abstracts and citations database, containing nearly 40,000 journal abstracts and citations from more than 7,000 publishers in 105 countries around the world (18). As an innovative information navigation tool, the search results of the Scopus database are more conducive to the evaluation of a certain field of literature.

### 2.2 Search design and data collection

Immune cells include B cells, T cells, NK cells, mononuclear macrophages, dendritic cells, granulocytes, and mast cells (19). We searched the database using the following query: (“single-cell DNA sequencing” OR “single-cell RNA sequencing” OR “single-cell sequencing”) AND (lymphocyte OR “B cell” OR “T cell” OR monocyte OR macrophage OR “dendritic cell” OR “NK cell” OR granulocyte OR mastocyte). The search was limited to articles published by December 31, 2021. After excluding non-English language articles, unpublished articles, errata, book chapters, and irrelevant literature, 1460 articles were collected and screened for analysis. All article field, including author, author ID, title, year, source, digital object identifier (DOI), country, language, article type and other information were exported in CSV file format.

### 2.3 Data analysis

The filtered database file included the fields of the authors, title, publication year, abstract, source journal, affiliation, DOI and keywords in each article. The file is opened by Microsoft Excel 2016 version for analysis. By reading the titles, abstracts, and the full text, if necessary, we categorized these articles by the research field and immune cell type. We screened out 284 cancer-related articles dealing with T cell research and imported them into Microsoft Excel 2016 version.

### 2.4 Visualization maps

GraphPad Prism 8 was used to produce a histograms of articles published by different countries each year from 2011 to 2021. The Draw Venn Diagram web page (http://bioinformatics.psb.ugent.be/webtools/Venn/) was used to create a Venn diagram of immune cells. VOSviewer 1.6.15.0 was used for country co-authorship, author co-authorship and author keyword co-occurrence analysis.

## 3 Results

### 3.1 Retrieval process and results

**Figure 1:**
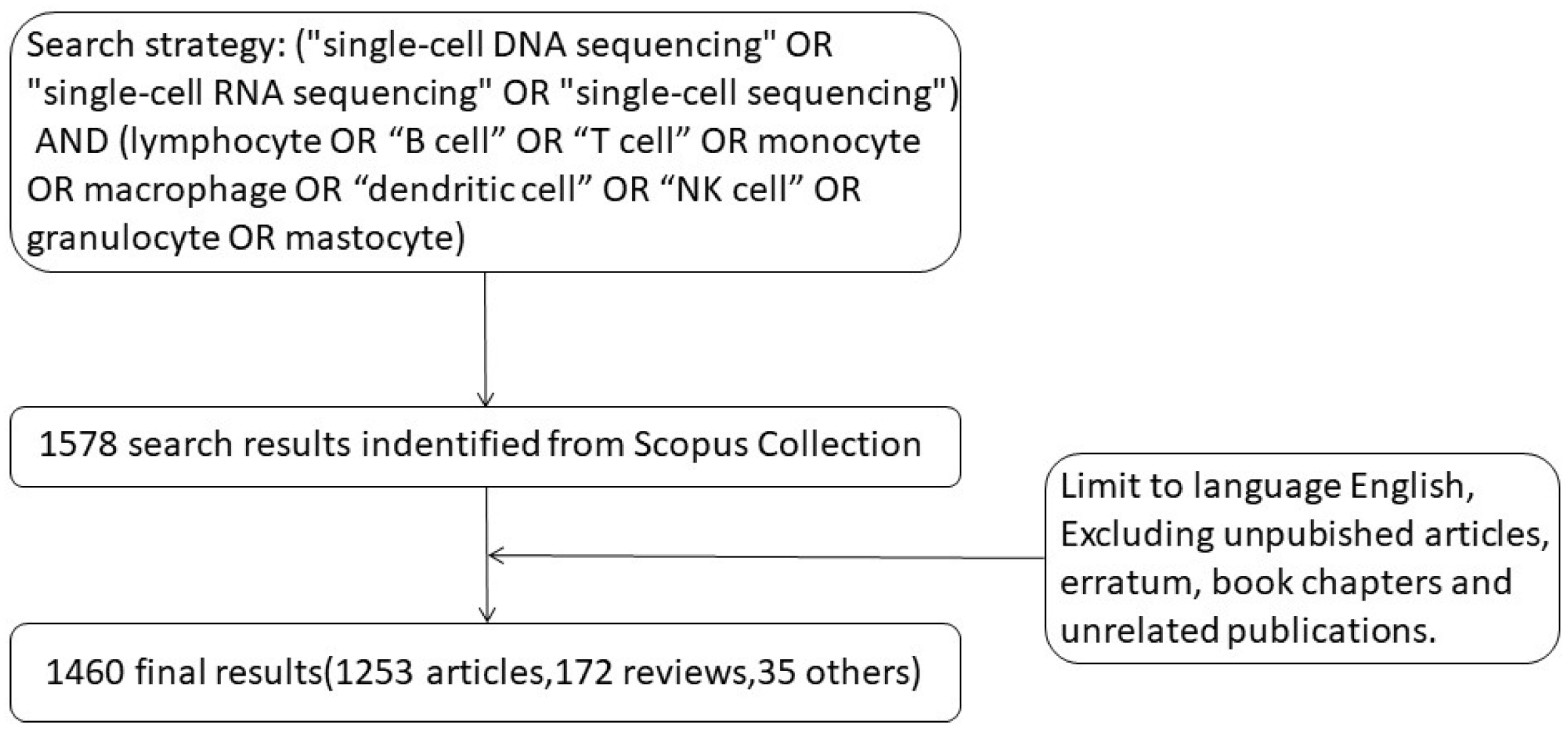
Data filtration processing and results. A total of 1578 results were found on Scopus using this search strategy. Non-English language articles, unpublished articles, errata, book chapters, and irrelevant literature were excluded. A total of 1460 articles were screened for analysis (including 1253 articles, 172 reviews, and 35 other types of articles).

We started with the search term [(“single-cell DNA sequencing” OR “single-cell RNA sequencing” OR “single-cell sequencing”) AND (lymphocyte OR “B cell” OR “T cell” OR monocyte OR macrophage OR “dendritic cell” OR “NK cell” OR granulocyte OR mastocyte)] and the title, abstract and keywords were searched in the Scopus database. By December 31, 2021, 1578 articles were retrieved. After applying the relevant exclusion criteria, 1460 articles were finally screened for follow-up analysis (**Figure 1**). The exclusion criteria were non-English language articles, unpublished articles, errata, book chapters, and irrelevant literature.All the 1460 articles (including 1253 articles, 172 reviews, and 35 other types of articles) were screened and included in the analysis.

### 3.2 Analysis of journals

The top 18 peer-reviewed journals that published the 1460 articles were ranked by number of publications. **Table 1** lists the top 18 journals with more than 15 publications, together with their impact factors. *Frontiers In Immunology* ranked first, with 113 articles and impact factor of 8.786. The second was *Nature Communications*, with 93 articles and impact factor of 17.694. Next was *Cell*, with 50 articles and impact factor of 66.85. Among these journals, the high impact factor journals included *Nature* (impact factor 69.504, 93 articles), *Science* (impact factor 63.714, 15 articles), *Immunity* (impact factor 43.474, 38 articles), *Science Immunology* (impact factor 39.630, 22 articles), *Nature Immunology* (impact factor 31.250, 25 articles). Among the 18 journals were publications on immunology, such as *Frontiers In Immunology*, *Immunity*, *Nature Immunology*, *Science Immunology*, and *Journal of Immunology*, as well as journals on cancer, such as *Frontiers in Oncology*, *Clinical Cancer Research*, and *Cancers*. The journal impact factor is an indicator of the academic value and research quality of the journal. Many publications on immune cells used in single-cell sequencing were published in journals of high impact factors, which indicates the value and importance of those researches. The types of journals also indicate the research hotspots were in the fields of immunity and oncology.

**Table 1:** Top 18 journals of the 1460 articles.

Rank^a^	Journal	Article number	Impact factor 2022^b^
1	Frontiers In Immunology	113	8.786
2	Nature Communications	93	17.694
3	Cell	50	66.850
4	Cell Reports	44	9.995
5	Immunity	38	43.474
6	Proceedings Of The National Academy Of Sciences Of The United States Of America	31	12.779
7	Nature	28	69.504
8	Nature Immunology	25	31.250
9	Science Immunology	22	30.630
10	Frontiers In Oncology	22	5.738
11	Genome Biology	21	17.906
12	Jci Insight	21	9.484
13	Scientific Reports	21	4.996
14	Clinical Cancer Research	19	13.801
15	Journal For Immunotherapy Of Cancer	17	12.469
16	Journal Of Immunology	17	5.426
17	Cancers	15	6.575
18	Science	15	63.714

^a^ Ranked by article number. Journals with the same number of articles were then ranked by impact factor.

^b^ Impact factor 2022 are metrics extracted from Scopus, introduced in detail in the Data Analysis section above.

### 3.3 Number of articles by year and country

**Figure 2:**
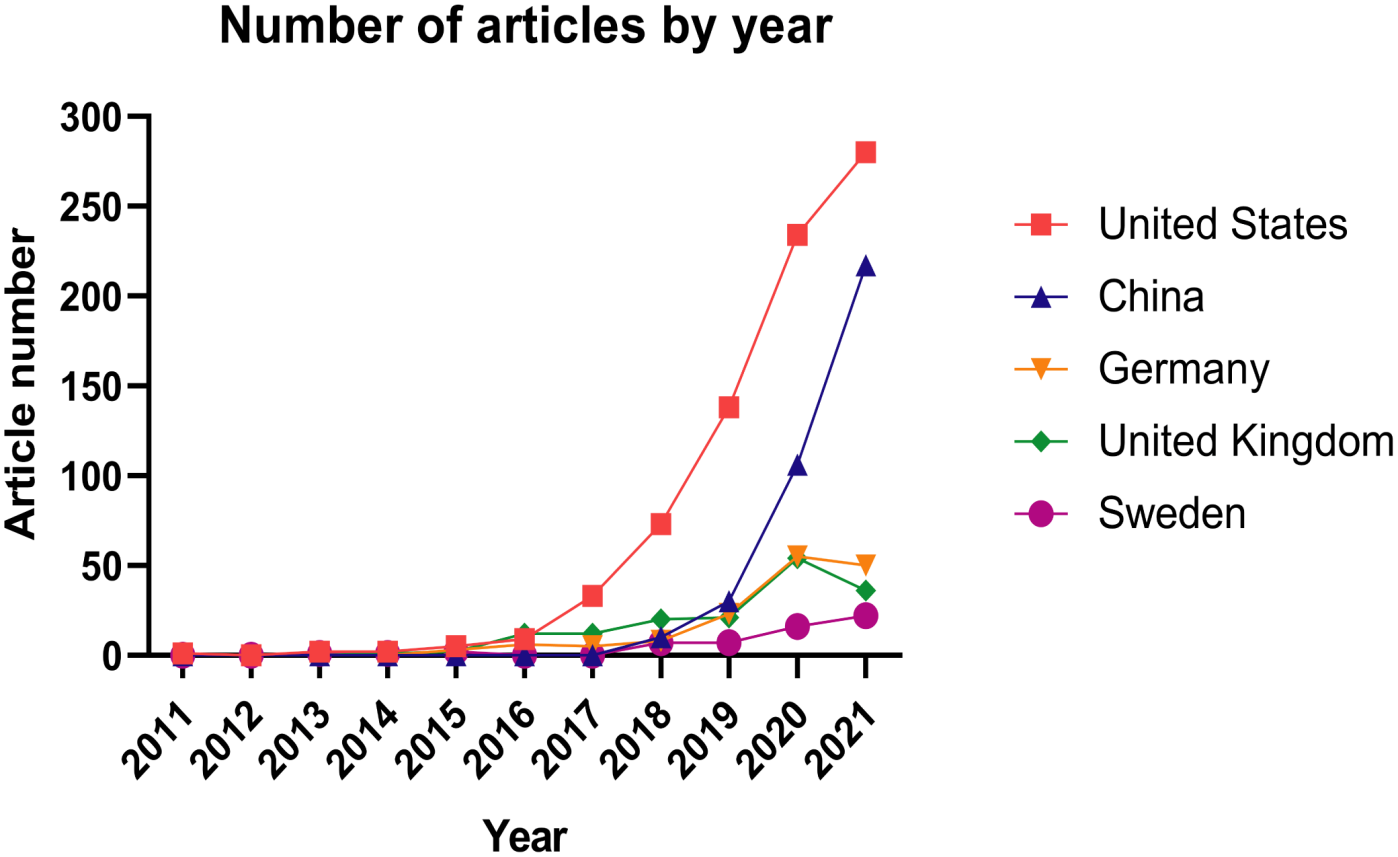
Number of articles published by different countries each year related to single-cell sequencing on immune cells. As shown in the icon, the United States is represented by the red line, China is represented by the blue line, Germany is represented by the yellow line, the United Kingdom is represented by the green line, and Sweden is represented by the purple line.

**Figure 2** shows the number of publications on immune cell-related research using single-cell sequencing according to the countries where research was conducted. The earliest study of single-cell sequencing of immune cells was an article published in 2011 by American researchers Surman et al. (6) on the immune response of CD8 lymphocytes to respiratory disease infection. No articles were published in 2012. In 2013, two articles were published one from the United States [single-cell RNA sequencing was used to study the different response of mouse bone marrow-derived dendritic cells to lipopolysaccharide (20)], while the second was jointly published by the United States, Sweden, Belgium, and Australia(application of single-cell sequencingto study CD4+ T cells after HIV infection (21)). The number of articles in the United States steadily increased since 2015; rising year by year since 2017, reaching 280 in 2021, reflecting the continued leadership of the USA in the field of immunology with single-cell sequencing. China’s research in this field started relatively late, with the first article published in 2018. However, in the past two years, thenumber of articles using immune cells and single-cell sequencing in China has increased dramatically, reflecting the rapid development of immunology-related single-cell sequencing in our country. The number of articles published in developed European countries such as Germany, the United Kingdom, and Sweden increased slowly from 2015 to 2020. Surprisingly, there was a sudden drop of articles published by German and United Kingdom bioscientists in 2021, which deserves further consideration. Overall, the more recent years have witnessed a marked increase in the number of articles related to single-cell sequencing of immune cells, suggesting that the technology has broad application prospects.

### 3.4 Author, keyword analysis of single-cell sequencing related immune cell research

**Figure 3:**
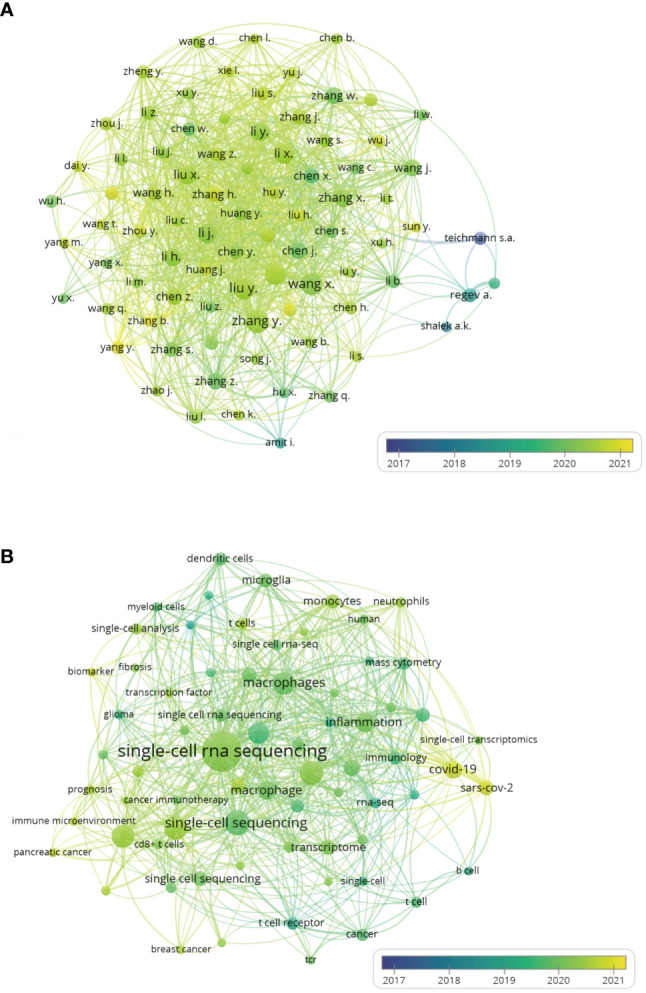
The author co-authorship and author keyword co-occurrence overlay visualization maps of single-cell sequencing publications on immune cells. **(A)** Author co-authorship overlay visualization maps. An author is represented by a circle, the number of publications of an author is represented by the size of the circle. The line represents the cooperative relationship between authors. The thickness of the line represents the degree of cooperation between authors. **(B)** Author keyword co-occurrence overlay visualization maps. The size of each circle indicates the frequency of occurrence of the author keyword. The distance between any two circles indicates their co-occurrence link, and the thickness of the connecting line indicates the link strength. The color of all circle is based on the average publication year.

VOSviewer was used to analyze author co-authorship, author keyword co-occurrence in the 1460 publications. In the author co-authorship visual map (**Figure 3A**), an author is indicated by a circle, the number of publications by that author is indicated by the size of the circle, the cooperative relationship between authors is represented by the lines. The degree of cooperation between authors is represented by the thickness of the line, and the average publication year is indicated by color. Authorswith more than 10 publications were selected, with a total of 83 authors in close collaboration. Teichmann appears blueviolet on the map, indicating the average publication year is 2017. The first paper by Teichmann and coworkers in this field was published in 2014 (9), in which they demonstrated by single-cell sequencing that Th2 cells produce the steroid pregnenolone in a helminth infection model. Teichmann is German and has published a total of 16 articles in this field, with a total link strength with other research groups of 11. Regev is from the United States, her name appears in turquoise on the map, which means the average publication year is 2018. Her first publication in the field was on mouse bone marrow-derived dendritic cells (21). She has published a total of 19 articles in this field, with the largest citations of 3189. The average publication year of Chinese papers is relatively late, but the Chinese authors were the top in terms of publication number and total link strength. As evident on the map, Zhang and coworkers published 46 articles with a total link strength of 140; while Liu et al. published 39 articles with a total link strength of 132.

Each article includes author keywords, and keyword co-occurrence analysis showed the subjects covered in single-cell sequencing-related immune cell research (**Figure 3B**). In this VOSviewer author-keyword co-occurrence visualization map the circle represents the frequency of the specific author-keywords, the line represents the total link strength, and the different colors represent the average publication year of the author keywords. Keyword “single-cell rna sequencing“ had the highest mention of 172 and the strongest total link strengths of 252. Its color is light green, suggesting that the average publication year is the first half of 2020. “scrna-seq”, “single-cell sequencing”, “single-cell RNA-seq”, “single-cell RNA-sequencing” are all different expression terms for “single-cell sequencing”, their occurrences were respectively 60, 67, 47 and 31, while their respective total link strengths were 80, 73, 44 and 36. Keywords related to T lymphocytes such as “t cells”, “t cell”, “cd8+ t cell”, “t cell exhaustion”, “thymus”, “tcr”, “t cell receptor”, “regulatory t cells” were used throughout the research periods, suggesting that T cells are the most interesting immune cells of single-cell sequencing throughout the study period. The keywords “macrophages” and “macrophage” were found in 90 and the total link strengths was 162, indicating that macrophages are also common immune cells in single-cell sequencing research. The circles of “macrophages” and “macrophage” are both light green in color, with an average publication year of 2019. “Dendritic cells”, “monocytes”, “neutrophils”, “myeloid cells” and “b cell” also appeared among the author-keywords on the map, but the scope of single-cell sequencing research on the above immune cells was not as broad as T cells and macrophages. “Glioma”, “pancreatic cancer”, “breast cancer”, “cancer” were all cancer-related author-keywords. The color of “glioma” was dark green, suggesting the publication year is earlier, while the yellow circles for “pancreatic cancer” and “breast cancer” suggest they were published later. This indicates that single-cell sequencing to detect immune cells in cancer, which focused on gliomas initially, is currently a hot area in pancreatic and breast cancer fields. On the right side of the map, two bright yellow circles, “covid-19” and “sars-cov-2”, were also evident, with occurrences of 32 and 23 and total link strength of 56 and 46, respectively. With the COVID-19 pandemic sweeping the world in 2020 (22), research on COVID-19 was carried out extensively, and single-cell sequencing technology is undoubtedly becoming a good research tool.

### 3.5 Research fields of immune cells-single-cell sequencing publications

By reading the abstracts of the 1460 articles and reviewing the full text if necessary, we divided the research fields of these documents into cancer, infection, cell function, autoimmunity, development, injury, metabolism, technology and others.

**Figure 4:**
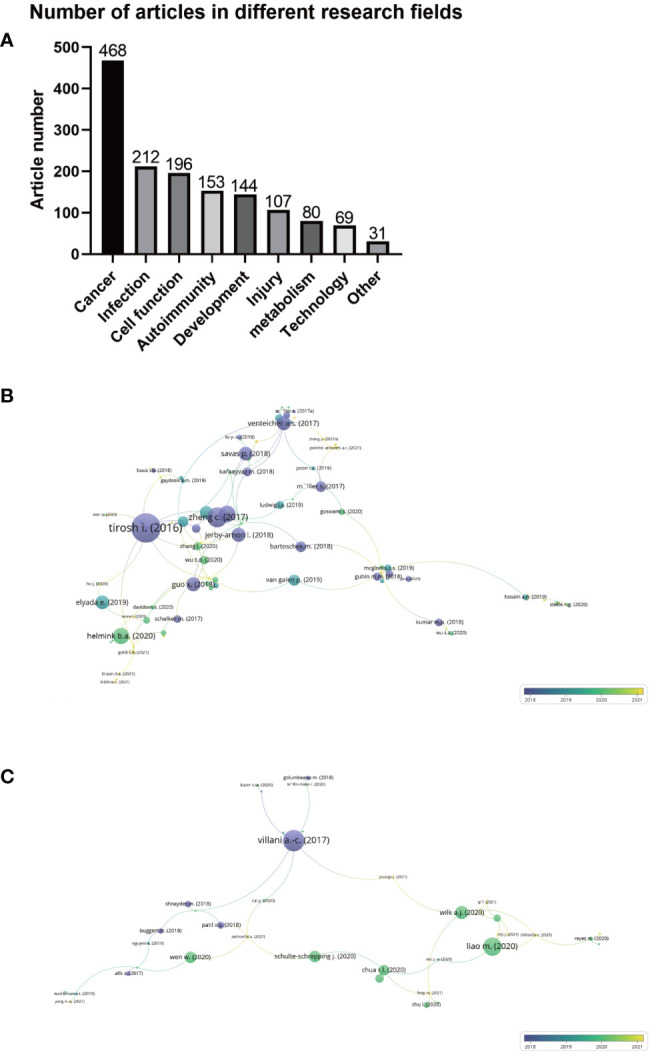
**(A)** Number of articles on single-cell sequencing of immune cells in different research fields. **(B)** Citation-documents analysis of articles using single-cell sequencing to detect immune cells in the field of oncology. **(C)** Citation-documents analysis of articles using single-cell sequencing to detect immune cells inthe field of infections. Circle size represents the number of citations, and lines represent the link strength of the documents with others.

**Figure 4A** shows the number of articles on single-cell sequencing of immune cells in different research fields. The largest number of articles dealing with single-cell sequencing of immune cells was in the field of cancer, with 468 items, accounting for 32.10% of the total count of articles. The type of cancer included glioma, colorectal cancer, lung cancer, breast cancer, pancreatic cancer, leukemia, ovarian cancer and others. These articles explored the pathogenesis of cancer by detecting immune cells, as well as the application of targeted therapy and immunotherapy in cancer. The second and third largest numbers of articles were infection and cell function, with 212 (14.52%) and 196 (13.42%) articles, respectively. Others included 153 (10.48%) articles on autoimmunity, 144 (9.86%) on development107 (7.33%) on injury, 80 (5.48%) on metabolism, 69 (4.73%) on technology, and 31 (2.12%) articlesin other fields. These findings suggest that single-cell sequencing has broad applications and promising prospects for immune cells.

Next, we conducted a citation-documents analysis of the top three research fields that used single-cell sequencing to detect immune cells, namely, cancer, infection, and cell function. As shown in **Figure 4B**, 108 of the 468 articles in the field of cancer were closely related, among which the most frequently cited article is the study of melanoma genotype and phenotype by Tirosh et al. published in 2016 (12), with 1365 citations, with a connection strength with other articles of 14. As shown in **Figure 4C**, of the 212 articles in the field of infection, 39 were closely related, among which the most frequently cited article was that of Villani and colleagues (23) published in Science in 2017, which studied the role of dendritic cells and monocytes in pathogen infection. This document was cited 893 times, and the connection strength with other articles was 9. Citation-documents analysis further illustrates that the hottest and most common field of single-cell sequencing on immune cells is cancer, followed by infection.

### 3.6 Immune cell types studied by single-cell sequencing technology

**Figure 5:**
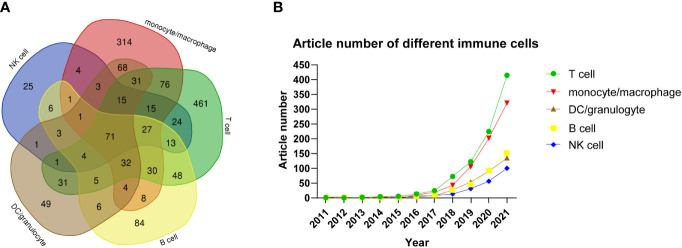
**(A)** Venn diagram of the number of articles on various types of immune cells studied by single-cell sequencing. **(B)** Article number of single-cell sequencing researches on different immune cells by year.

In **Figure 5A**, the Venn diagram presents the number of articles based on single-cell sequencing that evaluated various types of immune cells. Some articles used single-cell sequencing to study only one kind of immune cells, while others studied two or more types of immune cells. The number of articles that included T cells in single-cell sequencing studies was the largest, with a total of 884, of which 461 articles only studied T cells, accounting for 31.58% of the total number of articles. The remaining 423 articles studied other immune cells in addition to T cells. The second most popular immune cell used in single-cell sequencing was mononuclear macrophage, with a total of 700 articles, of which 314 used mononuclear macrophages only, accounting for 21.51%, while the remaining 386 articles used mononuclear macrophages and other immune cells. The numbers of articles using B cells, granulocytes/dendritic cells, and NK cells in single-cell sequencing were 84, 49, and 25, accounting for 5.75%, 3.36%, and 1.71%, respectively. Interestingly, 71 (4.86%) articlesanalyzed all the above-mentioned immune cellsin single-cell sequencing. These findings suggest that various immune cells are used in single-cell sequencing, among which T cells are the most popular, followed by mononuclear macrophages. We also analyzed the number of articles that used various immune cells in single-cell sequencing according to the year of publication. As shown in **Figure 5B**, from 2015 to 2021, the number of articles using various immune cells tended to increase, with those on T cells showing the fastest growth, followed by mononuclear macrophages. This illustrates the breakthrough of single-cell sequencing technology in the field of immunity.

### 3.7 Analysis of γδ T cell use in for single-cell sequencing studies

**Figure 6:**
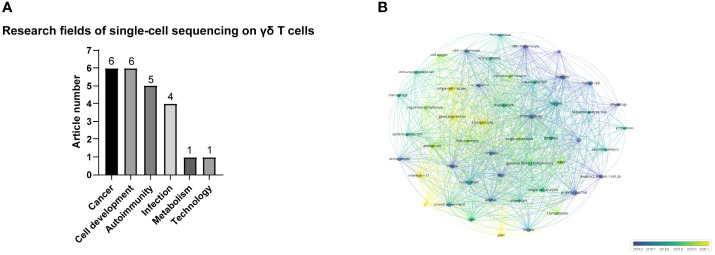
**(A)** Number of articles on different research fields involving γδ T cells and single-cell sequencing. **(B)** All Keywords co-occurrence analysis of single-cell sequencing on γδ T cells.

Among the articles on single-cell sequencing of T cells, 23 specifically mentioned γδ T cells. γδ T cells are T cells with innate immune functions, and their TCR consists of γ and δ chains (24). γδ T cells can be divided into different subgroups according to the composition of TCRγ or δ, such as Vδ1+, Vδ2+, Vδ3+γδ T (13). γδ T cells can also be divided into different subgroups according to their functions, such as γδ T cells that secrete IFN-γ and γδ T cells that secrete IL-17 (25). γδ T cells are involved in a variety of immune responses and immune regulation processes, and play important roles in resisting foreign infection, providing immunity against cancer and autoimmune diseases (26). Of the 23 articles of single-cell sequencing technology on γδ T cells, 6 articles were on cancer biology and γδ T cell development, 5 on autoimmune diseases, 4 on infection, 1 on metabolism and 1 article on new technology (**Figure 6A**). This demonstrates that single-cell sequencing studies of γδ T cells are more popular in the field of cancer biology and cell development. As shown in **Figure 6B**, “All Keywords co-occurrence analysis” was applied on these 23 articles. “All Keywords” included “Author Keywords” and “Index keywords”, providing more comprehensive information. A total of 51 keywords that appeared at least 5 times were selected. “Receptors” and “lymphocyte antigen receptor” appear in purple circles representing the early stage——June 2019. The yellow circles representing the late stage——January 2020 showed “gene expression”, “gene” and “interleukin 17.” These results indicate that over time, the focus of studies of γδ T cells with single-cell sequencing spanned from cell surface receptors to cell function.

### 3.8 Analysis of research using single-cell sequencing on DNA damage repair in cancer immunotherapy

**Figure 7:**
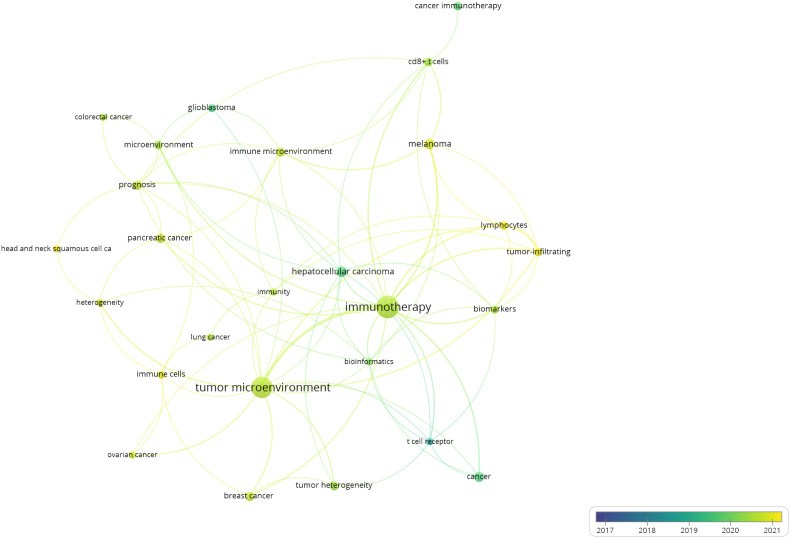
The author keyword co-occurrence overlay visualization maps of single-cell sequencing publications in cancer biology on T cell research. The circle size represents the frequency of use of the specified author keywords. The co-occurrence link is represented by the circle distance, and the strength of the link is indicated by the thickness of the connecting line. Circle color denotes the average publication year.

Based on the above stratification of 1,460 articles according to the types of immune cells and research fields, we manually selected all 284 articles that investigated T cells in cancer biology and performed the Author keyword co-occurrence analysisto detail the topics covered in this research field.As shown in **Figure 7** keyword “immunotherapy” was the largest mentioned with 43 and the strongest total link strength of 61. “Tumor microenvironment” followed, with 38 mention and total link strength of 51**Figure 7** also shows that the two keywords were both yellow-green, with average publication year being the second half of 2020. Thus, in recent years, immunotherapy and tumor microenvironment are hot trends in T cell research by single-cell sequencing in the field of cancer biology. Other cancer types identified in this analysis included “melanoma”, “hepatocellular carcinoma”, “pancreatic cancer”, “breast cancer”, “glioblastoma”, “colorectal cancer”, “colorectal cancer”, “lung cancer”, “head and neck squamous cell carcinoma” with a frequency mention of 10, 10, 8, 8, 5, 6, 5, 5, 5, respectively. This analysis demonstrates the wide application of single-cell sequencing for T cells in the generalized field of oncology.

Based on the above analysis that cancer immunotherapy is the hottest research field in single-cell sequencing of T cells, we focused next on DNA damage repair in cancer immunotherapy. DNA damage repair (DDR) gene is key in maintaining genome stability, and loss of DDR function may lead to the development and progression of cancers (27). In general, tumor immunogenicity includes three components (28): First, whether tumor cells produce neoantigens that can be recognized by the immune system. Second, whether tumor cells attract T cell infiltration and activation through the release of helper molecules, such as type 1 interferon and pro-inflammatory cytokines. Third, whether tumor cells can activate the killing function of T cells. DDR deficiency is an important determinant of tumor immunogenicity. Recent studies have shown that DDR is closely related to these three components. First of all, one of the main functions of DDR is to repair DNA damage and hence reduce existing DNA mutations, tumor mutational burden (TMB), number of neoantigen, and increase the sensitivity of tumors to immune checkpoint inhibitors (29). Second, the presence of extensive DNA damage in tumor cells due to DNA-damaging agents or endogenous deletion of DDR, leads to activation of the cGAS-STING signaling pathway, which may trigger type 1 interferon-mediated T cell activation (30). In addition, defects in DDR can also affect the expression of immune checkpoint proteins and co-stimulatory molecules, suggesting that defects in DDR can affect the interaction between T lymphocytes and tumor cells through a variety of pathways (31, 32). Targeting DNA damage–repair–related genes can enhance tumor immunogenicity and achieve successful treatment (33). For example, Zhe Tang et al. (34) treated mouse models of prostate cancer with a combination of DDR inhibitors and immune target blockers. They performed single-cell RNA sequencing of treated tumor tissues and found stronger innate immune activation and T cell-dependent treatment responses, as well as abundant apoptotic signal in the immune microenvironment. There are currently more than 80 clinical trials evaluating the efficacy of DDR-targeted therapies in combination with immune checkpoint inhibitors, and multiple independent early-stage clinical trials have shown that the combination of DDR-targeted therapies with immune checkpoint inhibitors is safe with encouraging antitumor effects (35).

Single-cell sequencing allows screening for higher-quality genomic information and explore the characteristics of immune cell infiltration in the tumor microenvironment with different DDR cell types. Lin et al. (27) divided liver cancer patients into DDR-activated subtype and DDR-suppressed subtype according to various clinical and molecular characteristics, and the results of single-cell sequencing showed a significant difference in the number of T cells between the two groups. Furthermore, Jin et al. (36) explored the relationship between repair of DNA damage and the immune microenvironment in small cell lung carcinoma. They found that homologous DNA pairing and chain exchange links in DNA double strand break homologous recombination repair correlated negatively with the immune microenvironment in small cell lung carcinoma. Inhibition of RAD51-mediated DNA pairing and chain exchange led to increased release of cytoplasmic double-strand (ds)DNA after DNA damage, thus activating the innate immune signaling pathway of cGAS-STING, promoting the expression of immune checkpoint–related molecules and the migration of peripheral blood mononuclear cells. This may have a positive effect in immunotherapy of small cell lung carcinoma, in conjunction with immune checkpoint therapy.

## 4 Discussion

In the past, the traditional sequencing results reflected only the genetic information of a cluster of cells, while ignoring the specific genetic information of a single cell (37). In order to overcome the above shortcomings of traditional sequencing, single-cell sequencing technology based on all-round, multi-layer and high-throughput has emerged. In 2009, Tang et al. (38) used the single-cell transcriptome sequencing (scRNA-seq) technology to detect the gene expression of mouse blastomere. With the emergence of new technologies, such as 10X Genomics, Drop-seq, Micro-well, and Split-seq, the cost threshold of single-cell sequencing has markedly diminished. Furthermore, single-cell sequencing technology was selected in 2013 as one of the excellent technologies of the year by Nature Methods (39). It is worth noting that single-cell Hi-C techniques to study the relationships of whole chromatin DNA at spatial locations on a genome-wide scale and multiomics single-cell sequencing that integrate epigenetics, transcriptomes and genotypes are not within our scope (40, 41).

Immune cells refer to those cells involved in or related to immune response, and include lymphocytes, dendritic cells, monocytes/macrophages, granulocytes and mast cells (42). The number of articles investigating immune cells by single-cell sequencing has grown significantly year by year, from 1 article in 2011 to 587 articles in 2021. The United States has published the earliest and the largest number of articles in this field, and has always maintained its leading position throughout the analysis period covered in this review. China has paid great attention to the blossom of single-cell technology, and proposed the “Thirteenth Five-Year Biotechnology Innovation Plan”, which included single-cell technology. This initiative was probably behind the rapid increase in the number of articles of single-cell sequencing on immune cells since 2018.

Reading the abstracts (full text of the articles if necessary) of the 1,460 published scientific papers identified cancer biology as the research field with the highest number of published articleson single-cell technology. Furthermore, keyword co-occurrence analysis showed that “glioma”, “pancreatic cancer”, “breast cancer” appeared frequently. Also, the glioma-related articles were published much earlierthan others. Using single-cell sequencing, Venteicher et al. (43) reported that the proportion of macrophages in the tumor microenvironment of gliomas increased with the severity of tumor malignancy. Our “Keyword co-occurrence Analysis” and Venn diagram showed that T lymphocytes were the most studied immune cells by single-cell sequencing. In June 2018, Professor Zhang Zemin’s research group (44) drew a T-cell immune map of lung and colorectal cancers at the single-cell level, outlining the subset classification, tissue distribution characteristics, intra-tumor population heterogeneity and drugs of T-cells in lung and colorectal cancers. In December 2018, Dr. Li Hanjie and colleagues from IdoAmit laboratory in Israel (45) drew a detailed immune cell map of melanoma through single-cell transcriptome sequencing and single-cell TCR sequencing analysis.

γδ T cells exist in small numbers but have unique functions (13). We analyzed 23 published articles of γδ T cell-single-cell sequencing analysis and found cancer biology and cell development to be the most popular research fields. According to “all keywords co-occurrence analysis”, the focus of single-cell sequencing with γδ T cells covered a wide topic from cell surface receptors to cell function. The detection of γδ T cells in melanoma biopsy samples by single-cell sequencing allowed Xiong et al. (46) to predict tumor response to immunotherapy and provide reference for clinical decision making. Furthermore, Chenet al. (47) accurately defined the time of ontogeny and the direction of functional differentiation by detecting γδ TCR through single-cell sequencing. Studies have shown that γδ T cells can exist in immunogenic mismatch repair-deficient colorectal cancer (48), and can be used as a tool to predict the response to immunotherapy (49), providing new insights for future research.

Through in-depth analysis of the single-cell sequencing articles on T cells in cancer biology, we found that immunotherapy and tumor microenvironment were research hotspots in recent years. Evidence suggests that weakening of the immune systemmay cause immune deficiency diseases or cancer (50). Previous studies confirmed immune system involvement in the development of most diseases, including cancers in different organs and even simple inflammatory reactions, but this often does not involve the behavior of a single cell line, but rather complex interaction of multiple cell lines (51). Therefore, comprehensive, multi-layer, high-throughput single-cell sequencing technology that can identify specific genetic information of individual cells provides a higher level of understanding of immunity involvement in diseases. For example, single-cell sequencing revealed that CD8+T cells with mutation-associated neoantigen specificity expressed lower levels of interleukin-7 receptor in non-small cell lung cancer treated with anti-PD-1, providing clues for overcoming PD-1 resistance (52).Furthermore, single-cell sequencing analysis also found that targeted repair of DNA damagecan coordinate synergistically or cooperatively with immunotherapy to produce effective anti-tumor response by regulating the immune microenvironment (34). Understanding T lymphocyte immune response mechanismsby single-cell sequencing technology has resulted in breakthrough in immunotherapy of related cancers, which also proves that single-cell sequencing can be used as a new and excellent tool in immune research (53).

Taken together,studies of single-cell sequencing on immune cells have developed rapidly in the past decades. Through the study of T cells by single-cell sequencing, scientists can explore in detail the complex interactions between cancer cells and immune cells in the tumor microenvironment, which provides an important basis for clinical immunotherapy, and can potentially lead to the development of new therapeutic strategies that can overcome tolerance of immunotherapy. In recent years, the continuous updating of biotechnology has improved tremendously the reliability of results obtained from single-cell sequencing and at the same time reduced the cost of this technology. The application of single-cell sequencing has increased progressively in the field of immunology, and it is hoped that T-cell immunotherapy of cancer further expands in the near future.

## Data availability statement

The original contributions presented in the study are included in the article/supplementary material. Further inquiries can be directed to the corresponding authors.

## Author contributions

YZ, YX and ZH preformed the bibliometric analysis and wrote the manuscript. JW, ZX, YM and GQ assisted in design the research and contributed to writing the manuscript. KC, WW and WM designed the research, organized the data statistics, and contributed to writing the the manuscript. All authors contributed to the article and approved the submitted version.
